# Whole-genome sequencing of feline calicivirus in domestic cats, South Korea, 2023

**DOI:** 10.3389/fvets.2025.1570761

**Published:** 2025-04-28

**Authors:** Ji-Heui Sohn, Dong-Yeop Lee, Tae-Hyeon Kim, Hyun-Jeong Sung, Hyomi Jang, Jung-Hyun Kim, Dong-Hun Lee

**Affiliations:** ^1^Department of Veterinary Internal Medicine, College of Veterinary Medicine, Konkuk University, Seoul, Republic of Korea; ^2^VIP Animal Medical Center, Seoul, Republic of Korea; ^3^Wildlife Health Laboratory, College of Veterinary Medicine, Konkuk University, Seoul, Republic of Korea; ^4^Avian Disease Laboratory, College of Veterinary Medicine, Konkuk University, Seoul, Republic of Korea

**Keywords:** feline calicivirus, phylogenetic analysis, reverse transcription polymerase chain reaction, virulent systemic disease, whole-genome sequencing

## 1 Introduction

Feline calicivirus (FCV) is a prominent infectious pathogen of cats (1–4). Classical FCV causes mild symptoms, such as respiratory disease and oral inflammation, which tend to be self-limiting and are referred to as oral respiratory diseases (ORD) (4). However, virulent systemic feline calicivirus (VS-FCV) causes virulent systemic disease (VSD) and high mortality. This highly virulent form has spread to Europe, the United States of America, and, more recently, China and South Korea (5–11). Although sporadic clinical cases have been reported in South Korea, there is a lack of genetic analysis based on whole-genome sequencing (WGS) of VS-FCV isolated from these cases (10, 12).

FCV is a single-stranded (1), positive-sense, non-enveloped RNA virus of the genus *Vesivirus*, family *Caliciviridae* (2, 3). The FCV genome encodes three open reading frames (ORFs): ORF1 for nonstructural proteins, ORF2 for the major capsid protein VP1, and ORF3 for the minor capsid protein VP2 (13). FCV exhibit considerable genetic and antigenic diversity, with region E, comprising amino acids (aa) 426–521 in the protruding domain of VP1, being the primary contributor to this variability. FCV vaccines have been available for over four decades; however, they do not provide complete immunity, leading to the widespread distribution of diverse FCV strains (14).

In this study, oropharyngeal swab samples from FCV infected cats were collected from an animal hospital in South Korea and analyzed for their whole genome sequences using multiplex tiling reverse transcription polymerase chain reaction and Illumina next-generation sequencing.

## 2 Materials and methods

### 2.1 Primer design and tiling amplicon PCR for whole genome sequencing

A multiplex tiling RT-PCR approach was developed to assess the full viral genome coverage of clinical samples. Initially, all available FCV genomes were downloaded from the National Center for Biotechnology Information database and aligned to generate a consensus sequence. Based on this, three pairs of primers were designed using PrimalScheme software, amplifying the entire genome of 7,800 bp with ~100–200 bp overlaps (15) (Table 1). Oropharyngeal swabs from six cats were subjected to RT-PCR using the OneStep RT-PCR Kit (Qiagen, Hilden, Germany) following the manufacturer's instructions. The PCR mixture was prepared by mixing 10 ul 5x QIAGEN One-Step RT-PCR buffer, 10 ul dNTP mix, 0.6 uM of each primer, 2 ul of template RNA, and 31 ul nuclease-free water. PCR amplification conditions were: 45°C for 30 min, 95°C for 15 min, followed by 40 cycles of 94°C for 30 s, 59 (primer set 1)/65 (primer set 2, 3)°C for 1 min, and 68°C for 3 min. The PCR products were then visualized via electrophoresis on 1% agarose gels, showing ~1.3–2.9 kbp amplicons. PCR products were pooled, and ~100,000 next-generation sequencing (NGS) reads of 150 bp per sample were produced using Illumina DNA prep kit and Nextseq 500 NGS system (Illumina, USA) according to the manufacturer‘s instructions to achieve >1,000 × genome coverage.

**Table 1:** Tiling amplicon PCR primer sets used for amplification of feline calicivirus genome.

**Primer (nt position)**	**Primer sequences (5'-3')**	**Product length (bp)**	**Position (nt)**
FCV-1 For (1–23)	GTAAAAGAAATTTGAGACAATGT	2,557	1–2,557
FCV-1 Rev (2,541–2,557)	AGCACATCATATGCGGC		
FCV-2 For (2,428–2,446)	CTACCCGCCAATCARCATG	2,925	2,428–5,353
FCV-2 Rev (5,335–5,353)	ACGTTAGCGCAGGTTGAGC		
FCV-3 For (5,306–5,328)	ACTGTGATGTGTTCGAAGTTTGA	2,494	5,306–7,800
FCV-3 Rev (7,785–7,800)	CCCTGGGGTTAGGCGC		

### 2.2 Genome assembly and phylogenetic analysis

The raw reads obtained from NGS were subjected to trimming and filtering to remove adapters and low-quality bases using BBDuk (v38.84) with a minimum quality threshold set at 30 and a minimum length of 50 bp. Subsequently, *de novo* and reference-based assemblies of the genome sequences were conducted using Geneious Prime version 2024.0.4 (Biomatters Ltd., Auckland, New Zealand). For reference-based assembly, the trimmed reads were mapped to the KP361 viral genome (GenBank accession number: MZ542330) using Geneious Mapper with default options.

To perform genetic analysis, 27 FCV sequences were downloaded from the NCBI GenBank database and aligned using MAFFT Multiple Sequence Alignment software (v7.490) (16). Maximum likelihood (ML) phylogenetic analysis was performed using RaxML (v8.2.13) with a GTR GAMMA model and a rapid bootstrapping option set to 1,000 replicates (17).

To further investigate the possible genetic relationship between our strains and VS-FCVs, the inferred amino acid (aa) sequences of the hypervariable region E of the viruses were mapped to identify seven aa residue positions (438, 440, 448, 452, 455, 465, and 492), whose physical and chemical properties were previously shown to be statistically significant for differentiation between the upper respiratory tract and VS-FCV pathotypes (Supplementary Tables S1, S2) (18).

A recent study identified two linear epitopes within the P2 sub-domain of VP1, specifically in the E5 antigenic hypervariable region. The first epitope, spanning aa 431–435 (PAGDY), is highly conserved and induces a non-neutralizing immune response. The second epitope, located at aa 445–451 (ITTANQY), is highly variable and elicits a neutralizing immune response (19). We comparatively analyzed the linear epitope sequences of the viruses from this study and other previously reported viruses.

## 3 Descriptive results

In 2023, a total of six cats presented to the clinic with clinical symptoms and tested positive for calicivirus via real-time RT-PCR. Each cat exhibited systemic symptoms, including tongue and skin ulcers, respiratory distress, anorexia, fever, anemia, and pleural effusion. Of the six cats, three survived, whereas the other three died. All cats completed their primary vaccination series early in life and received at least one booster vaccination. Based on molecular FCV detection and clinical findings, we presumptively diagnosed VS-FCV infection. However, systemic infection was not definitively confirmed in these cases, which represents a limitation, as it does not fully meet the established criteria for VS-FCV.

**Figure 1:**
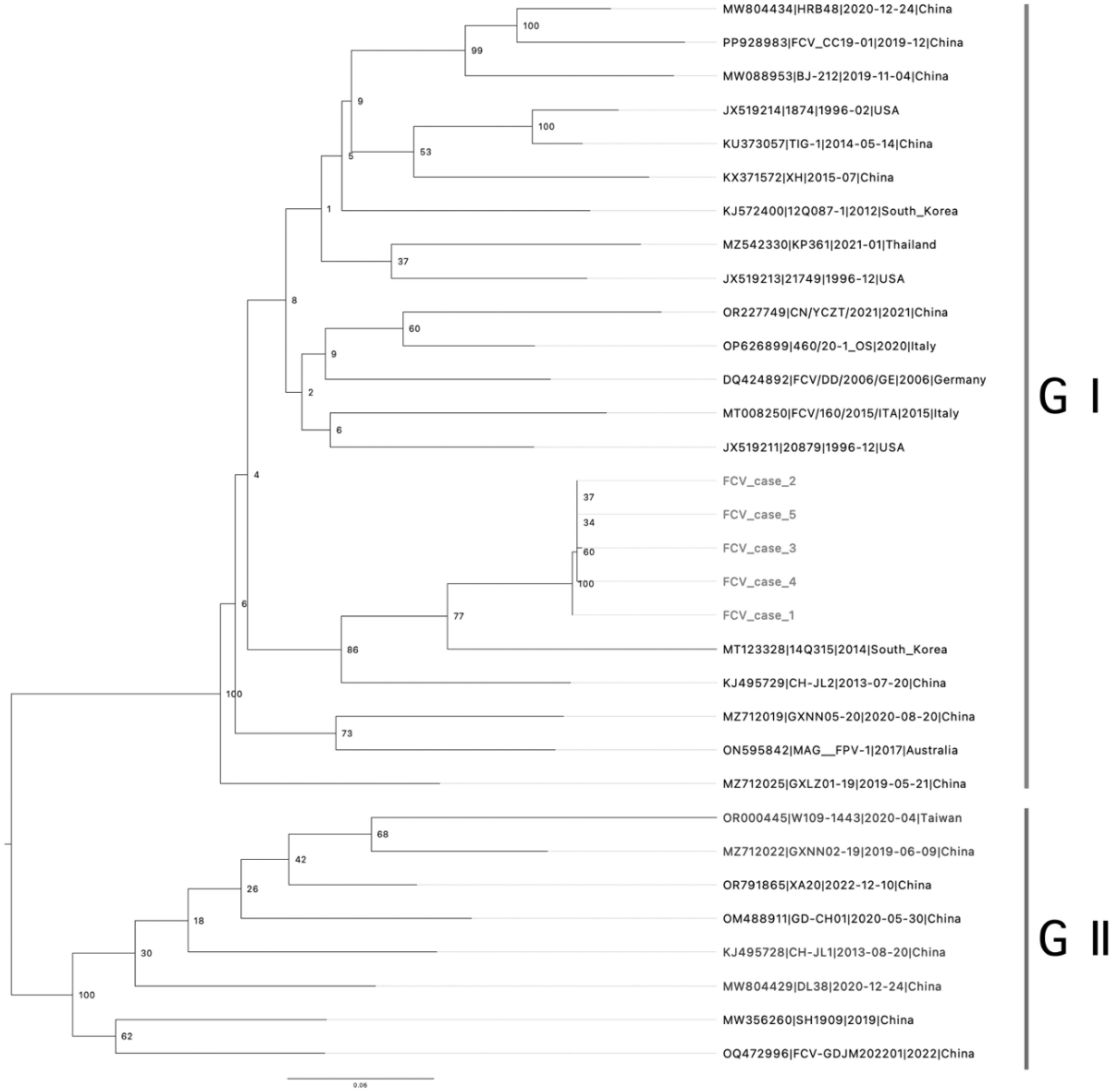
Phylogenic tree of viral protein 1 (VP1) capsid protein: phylogenetic analysis based on the amino acid sequence of the full-length VP1 capsid protein was generated using the maximum likelihood, supplying statistical support with bootstrapping of 1,000 replicates. Rooted to midpoint. The scale bars show the number of substitutions per site. The numerical values represent 1,000 bootstrap replicate values expressed as a percentage. Genotypes are indicated by vertical bars.

The tiling amplicon PCR method has demonstrated remarkable efficiency and productivity in generating complete viral genome sequences directly from clinical samples (15). In this study, we developed multiplex tiling RT-PCR primer panels and successfully obtained the complete genomes of VS-FCVs directly from the clinical samples of five cats using the newly designed primer set. The sample obtained from cat 6 contained insufficient sequencing material. The samples obtained from cat 1–5 shared a high nucleotide sequence identity of >99.8%. Based on the phylogenetic analysis of the VP1 gene, FCV has been categorized into two genotypes: GI and GII (20). The GI genotype is globally dominant and widespread, whereas all identified GII strains have been isolated in Asia. The ML phylogenetic analysis showed that the FCVs sequenced in this study formed a monophyletic clade and belonged to the genogroup I (Figure 1). They showed the closest genetic relationship with an FCV strain identified in South Korea in 2014 (GenBank Accession no. MT123328; nucleotide sequence identity: 85.05–85.22%). However, they formed a long branch in the ML phylogeny, due to the scarcity of FCV genome sequences.

The high degree of genomic plasticity in FCV led to the emergence of various variants, some of which are associated with severe clinical diseases. To further investigate the possible genetic relationship between our FCVs and other VS-FCVs, the deduced aa sequences of the hypervariable region E were mapped to compare seven aa residue positions (438, 440, 448, 452, 455, 465, and 492), whose physical and chemical properties were previously shown to be statistically significant for differentiation between the ORD and VSD-FCV pathotypes (18). In our analysis, the predicted properties for virulent pathotypes were observed in four of the seven residues (positions 448, 452, 455, and 492) in the hypervariable region E (Supplementary Table S1). We assume that these VSD markers may not reliably distinguish between current VSD and less pathogenic viruses in South Korea. Further research is required to establish a clear, stringent differentiation between VSD and ORD FCV strains clinically and at the molecular level (2).

We conducted a comparative analysis of the linear epitope sequences in VP1 and found significant sequence differences in the neutralizing epitope (aa 445–451) among the FCV strains. The pairwise identity of the neutralizing epitope was relatively lower (52.0%) than 87.5% of the non-neutralizing epitope and 86.7% for the entire VP1 (Supplementary Table S2). We assume that these genetic variations may result in vaccine-generated antibodies being less effective at neutralizing the virus during actual infections. Some endemic calicivirus strains occurring sporadically have shown genetic diversity compared with vaccine strains (21, 22), and reports have revealed the emergence of vaccine-resistant viruses (23, 24).

Given the recent reports of VS-FCV infections and outbreaks resulting in high virulence in cats within veterinary hospitals, complete genome sequencing of suspected cases would be helpful in monitoring the evolution and transmission of FCVs. The multiplex tiling RT-PCR and NGS approach used in this study demonstrates significant potential for integration into diagnostic workflows, providing a rapid and reliable method for complete genome sequencing and molecular epidemiological investigation of FCV from clinical samples. This study highlights the need to develop more effective vaccines based on genomic surveillance data to address the diverse and rapidly evolving strains of FCV.

## Data Availability

The genome sequences generated in this study can be found in the GenBank under accession numbers PV054606-PV054611.
