# Supplementary material for: Whole-genome sequencing of feline calicivirus in domestic cats, South Korea, 2023
Source: Front Vet Sci. 2025 Apr 28;12:1570761. doi: 10.3389/fvets.2025.1570761 (PMC12066559; doi:10.3389/fvets.2025.1570761)
Supplement: Supplementary file 1 [file Table_1.docx]

# Supplemental Tables

**Table S1.** Amino acid residues of region E of the viral protein 1 capsid protein associated with the virulent systemic feline calicivirus pathotype.

| Virus | Amino acid position and characteristics* | | | | | | | M/T |
| --- | --- | --- | --- | --- | --- | --- | --- | --- |
|  | 438 | 440 | 448 | 452 | 455 | 465 | 492 |  |
|  | Non-Polar,  Aliphatic Chain | Non-Small | Polar, Positive Charged | Non-Small | Non-Negative | Polar | Small |  |
| FCV_case_1 | T | S | **K** | **E** | **T** | G | **V** | 4/7 |
| FCV_case_2 | T | S | **K** | **E** | **T** | G | **V** | 4/7 |
| FCV_case_3 | T | S | **K** | **E** | **T** | G | **V** | 4/7 |
| FCV_case_4 | T | S | **K** | **E** | **T** | G | **V** | 4/7 |
| FCV_case_5 | T | S | **K** | **E** | **T** | G | **V** | 4/7 |
| Vaccine/F9/USA/1960/CAA77636 | T | G | A | D | D | G | L | 0/7 |
| ORD/127/USA/2004/ABI84196 | T | G | A | D | D | **S** | R | 1/7 |
| ORD/12Q087-1/South-Korea/2012/AIA09956 | T | S | A | D | D | G | **V** | 1/7 |
| ORD/12Q087-5/South-Korea/2012/AIA09959 | T | G | A | D | D | G | **V** | 1/7 |
| ORD/131/USA/2004/ABI84198 | T | G | A | D | D | G | **V** | 1/7 |
| ORD/796/USA/2003/ABI84200 | T | **E** | G | D | **R** | G | L | 2/7 |
| ORD/CH-JL4/China/2015/ALO69832 | T | D | A | D | **S** | **S** | I | 2/7 |
| ORD/DD2006/Germany/2006/ABD84433 | T | G | A | D | D | G | **V** | 1/7 |
| ORD/FB-NJ-13/China/2013/AIS22460 | T | **Q** | A | D | **T** | **S** | L | 3/7 |
| ORD/Gon/Japan/2003/AHZ59401 | T | **Q** | P | N | **T** | **S** | L | 3/7 |
| ORD/HB-S4/China/2014/ALI87297 | T | S | A | D | D | G | I | 0/7 |
| ORD/HRB-SS/China/2014/AII00833 | T | G | A | D | D | G | **V** | 1/7 |
| ORD/ITO/Japan/2002/AHZ59402 | T | S | A | D | D | G | **V** | 1/7 |
| ORD/NH10/USA/2009/AAY44310 | T | G | A | D | **S** | G | **V** | 2/7 |
| ORD/NH12/USA/2009/AAY44312 | T | G | A | D | D | G | **V** | 1/7 |
| ORD/NH3/USA/2000/AAT66084 | T | G | G | D | D | **S** | R | 1/7 |
| ORD/NH4/USA/2009/AAY44304 | T | G | P | D | **V** | G | R | 1/7 |
| ORD/NH5/USA/2009/AAY44305 | T | G | A | D | D | G | R | 0/7 |
| ORD/NH6/USA/2009/AAY44306 | T | **R** | A | D | D | G | L | 1/7 |
| ORD/NH7/USA/2009/AAY44307 | **V** | **Q** | **K** | **E** | **T** | **S** | **V** | 7/7 |
| ORD/NH8/USA/2009/AAY44308 | T | **R** | **K** | **E** | D | G | **V** | 4/7 |
| ORD/NH9/USA/2009/AAY44309 | T | G | A | D | E | G | **V** | 1/7 |
| ORD/SH-2014/China/2014/ALM55428 | T | G | **K** | **E** | D | **S** | I | 3/7 |
| ORD/TFHLJ-8/China/2013/AIN37114 | T | S | A | D | D | **S** | K | 1/7 |
| ORD/um3/Japan/2001/AHZ59403 | **I** | **Q** | G | D | **T** | **S** | I | 4/7 |
| VSD/ARI/USA/1998/ABI84212 | **V** | G | **K** | **E** | D | G | **V** | 4/7 |
| VSD/Deuce/USA/2004/ABI84202 | T | **Q** | P | D | **I** | G | R | 2/7 |
| VSD/Georgie/USA/2003/ABI84206 | T | G | G | D | D | **S** | **V** | 2/7 |
| VSD/H1/USA/1999/AAT66087 | **V** | **Q** | **K** | **E** | **T** | **S** | **V** | 7/7 |
| VSD/H2/USA/2002/AAT66090 | T | G | **R** | **E** | D | **S** | **V** | 4/7 |
| VSD/Kaos/USA/2002/ABI84214 | **V** | G | **K** | **E** | **T** | **S** | **V** | 6/7 |

*Residues in bold indicate amino acids matching with the VSD configuration.

M/T, matched/total; ORD, oral respiratory disease; VSD, virulent systemic disease

**Table S2**. Amino acid sequences of the E5 hypervariable region of feline calicivirus strains.

| Sample | pathotype | aa 431-435 | aa 445-451 |
| --- | --- | --- | --- |
| FCV_case_1 | - | PAGNY | IVTKAGY |
| FCV_case_2 | - | PAGNY | IVTKAGY |
| FCV_case_3 | - | PAGNY | IVTKAGY |
| FCV_case_4 | - | PAGNY | IVTKAGY |
| FCV_case_5 | - | PAGNY | IVTKAGY |
| Vaccine/F9/USA/1960/CAA77636 | Vaccine | PAGDY | ITTATGY |
| ORD/127/USA/2004/ABI84196 | ORD | PAGNY | ITTAAQY |
| ORD/12Q087-1/South-Korea/2012/AIA09956 | ORD | PAGDY | ITTAAKY |
| ORD/12Q087-5/South-Korea/2012/AIA09959 | ORD | PAGDY | IVTAAKY |
| ORD/131/USA/2004/ABI84198 | ORD | PAGDY | ITTASEY |
| ORD/796/USA/2003/ABI84200 | ORD | PVGNY | ITTGEDY |
| ORD/CH-JL4/China/2015/ALO69832 | ORD | PAGNY | IITASQY |
| ORD/DD2006/Germany/2006/ABD84433 | ORD | PAGDY | ITTAQVF |
| ORD/FB-NJ-13/China/2013/AIS22460 | ORD | PAGDY | ITTAMEY |
| ORD/Gon/Japan/2003/AHZ59401 | ORD | PAGDY | ITTPSGY |
| ORD/HB-S4/China/2014/ALI87297 | ORD | PAGDY | IVTATEY |
| ORD/HRB-SS/China/2014/AII00833 | ORD | PAGDY | IITANAY |
| ORD/ITO/Japan/2002/AHZ59402 | ORD | PAGDY | ITTAADY |
| ORD/NH10/USA/2009/AAY44310 | ORD | PAGNY | ITTAKEY |
| ORD/NH12/USA/2009/AAY44312 | ORD | PAGDY | IVTAREF |
| ORD/NH3/USA/2000/AAT66084 | ORD | PSGDY | IITGADY |
| ORD/NH4/USA/2009/AAY44304 | ORD | PAGDY | ITTPKEY |
| ORD/NH5/USA/2009/AAY44305 | ORD | PSGDY | IITASSF |
| ORD/NH6/USA/2009/AAY44306 | ORD | PAGDY | IITAAEY |
| ORD/NH7/USA/2009/AAY44307 | ORD | PAGDY | IVTKRGY |
| ORD/NH8/USA/2009/AAY44308 | ORD | PAGDY | IRTKAEY |
| ORD/NH9/USA/2009/AAY44309 | ORD | PAGDY | ITTAAGY |
| ORD/SH-2014/China/2014/ALM55428 | ORD | PAGDY | IATKQQY |
| ORD/TFHLJ-8/China/2013/AIN37114 | ORD | PAGNY | IATATEY |
| ORD/um3/Japan/2001/AHZ59403 | ORD | PAGDY | ITTGESF |
| VSD/ARI/USA/1998/ABI84212 | VSD | PAGDY | ITTKDKY |
| VSD/Deuce/USA/2004/ABI84202 | VSD | PAGDY | IVTPSRF |
| VSD/Georgie/USA/2003/ABI84206 | VSD | PAGDY | ITTGEKY |
| VSD/H1/USA/1999/AAT66087 | VSD | PAGDY | IATKQAY |
| VSD/H2/USA/2002/AAT66090 | VSD | PAGKY | ILTRNDY |
| VSD/Kaos/USA/2002/ABI84214 | VSD | PAGDY | IATKAQY |
| Pairwise identity |  | 87.5% | 52.0% |

aa, amino acid; FCV, feline calicivirus; ORD, oral respiratory disease; VSD, virulent systemic disease
